# The emerging risk of oropharyngeal and oral cavity cancer in HPV-related subsites in young people in Brazil

**DOI:** 10.1371/journal.pone.0232871

**Published:** 2020-05-14

**Authors:** Fabrício dos Santos Menezes, Maria do Rosário Dias de Oliveira Latorre, Gleice Margarete de Souza Conceição, Maria Paula Curado, José Leopoldo Ferreira Antunes, Tatiana Natasha Toporcov

**Affiliations:** 1 Department of Health Education, Federal University of Sergipe, Lagarto, Sergipe, Brazil; 2 Department of Epidemiology, School of Public Health, University of São Paulo, São Paulo City, São Paulo State, Brazil; 3 Department of Epidemiology, International Research Center, A.C. Camargo Cancer Center, São Paulo City, São Paulo State, Brazil; 4 International Prevention Research Institute, Ecully, France; Georgetown University, UNITED STATES

## Abstract

Human papillomavirus (HPV) is responsible for the rise in the incidence of cancer in the oropharynx, tonsils, and base of the tongue (i.e., HPV-related subsites). HPV triggered the changes in the epidemiology of oropharyngeal and oral cavity cancer (OPC/OCC) in Asia, Europe, North America, and Oceania. Hence, the incidence of cancer in HPV-related subsites is augmenting, while that in other HPV-unrelated subsites is decreasing. In South America, although the incidence of HPV-positive tumors has gradually increased, there is an atypically low prevalence of HPV in people with OPC/OCC. To clarify whether this dramatic shift in incidence trends also occurred in this population, we estimated the burden of HPV on the incidence trends of OPCs/OCCs in São Paulo city in Brazil. In this population-based study, we categorized OPCs/OCCs by HPV-related and HPV-unrelated subsites. We used Poisson regression to assess the age-standardized incidence rates (ASRs) stratified by sex and age groups, as well as to examine the age-period-cohort effects. There were 15,391 cases of OPCs/OCCs diagnosed in HPV-related (n = 5,898; 38.3%) and HPV-unrelated (n = 9,493; 61.7%) subsites. Overall, the ASRs decreased for most subsites, for both sexes and for all age groups, except for HPV-related OPC/OCC in young males and females, which increased by 3.8% and 8.6% per year, respectively. In the birth-cohort-effect analysis, we identified an increasing risk for HPV-related OPC/OCC in both sexes in recent birth cohorts; however, this risk was sharply decreased in HPV-unrelated subsites. Our data demonstrate an emerging risk for HPV-related OPC/OCC in young people, which supports prophylactic HPV vaccination in this group.

## Introduction

Human papillomavirus (HPV) is an oncogenic virus that is sexually transmitted, and it is the cause of an estimated 630,000 new cancers each year worldwide [1]. There is a causal association between HPV and oropharyngeal and oral cavity cancers (OPCs/OCCs) [2]. In addition to the unique carcinogenesis pathway [3] and HPV DNA identified in tumors [4], HPV-positive OPCs/OCCs are associated with clinical and epidemiological features, such as a young age at diagnosis, high-risk sexual behaviors [5,6], and enhanced survival rates [7], which distinguish these cancers from their HPV-negative counterparts.

In the mid-2000s, there was a striking rise, from 40.5% to 72.2%, in the overall HPV prevalence of oropharyngeal tumors [4]. Likewise, oropharyngeal and oral cavity subsites strongly associated with HPV infection (hereafter referred to as HPV-related subsites) have increased in both sexes in Asia [8], Oceania [9,10], and North America [11–13], whereas HPV-unrelated subsites have decreased, similar to the prevalence of smoking [14] and lung cancer incidence [15]. There is a global concern regarding the changing epidemiology of OPC/OCC. However, little information is available on the burden of this “virus-related cancer epidemic” in South America [16]; this information is urgently needed because South America has the highest prevalence of oral HPV in healthy individuals worldwide [17]. In Brazil, studies found a low prevalence of HPV in oropharynx tumors ranging from 4.4% to 8.8% [18–20]. Conversely, patients from a private hospital had an HPV positivity rate of 59.1% [21], which demonstrates the HPV heterogeneity in the country. Due to these discrepancies in the prevalence of HPV, it is crucial to monitor these subsites to clarify whether this dramatic shift in incidence trends observed worldwide also affects this population.

To the best of our knowledge, no population-based study has focused on the burden of HPV associated with OPC/OCC incidence in Brazil. Prophylactic HPV vaccinations were introduced in 2014 in an effort to hasten a decrease in HPV prevalence, but its need in oral cavity and oropharyngeal subsites at the population level was unknown. For these reasons, we assessed the incidence trends of OPC/OCC in HPV-related and HPV-unrelated subsites over the 15 years prior to large-scale HPV immunization in São Paulo city.

## Materials and methods

### Data source

The Research Ethics Committee (nº 83218318.8.0000.5421) and the Technical Advisory Committee of the Population-based Cancer Registry in the city of São Paulo (RCBP-SP) approved our investigation. We accessed data on the RCBP-SP repository in July 2018 [22]. All datasets were fully anonymized before using them.

This population-based study included new cases of OPC/OCC diagnosed between 1997 and 2013 in the RCBP-SP database. In São Paulo city, the RCBP-SP records cancer data of more than 12 million residents from 301 sources, including hospitals, clinics, and death investigation services; this database is one of the oldest and largest cancer registries in Latin America [23].

### Classification of anatomic sites

To investigate the potential role of HPV in the burden of OPC/OCC, we classified anatomical codes as a proxy for HPV exposure based on robust scientific evidence [4,8,24–27] because cancer registries do not contain information on HPV DNA testing from tumors [25,28,29]. Hence, HPV-related subsites comprised individuals with the following subsites: base of the tongue (C01), tonsil (C02.4, C09.0-C09.1, and C09.8-C09.9), oropharynx (C10.0-C10.4 and C10.8-C10.9), soft palate and uvula (C05.1-C05.2), and Waldeyer’s ring (C14.2). Likewise, HPV-unrelated subsites comprised individuals with other parts of the tongue (C02.0-C02.3 and C02.8-C02.9), gums (C03.0-C03.1 and C03.9), mouth (C04.0-C04.1, C04.8-C04.9, C06.0-C06.2, and C06.8-C06.9), and hard palate (C05.0, and C05.8-C05.9). According to Chaturvedi et al. (2013), we included all histological codes because approximately 95% of head and neck cancers are squamous cell carcinomas [15].

### Statistical analyses

The incidence rates were further age-standardized per 100,000 persons using the direct method, the World Health Organization (WHO) standard population [30], and data provided by the SEADE Foundation [31].

For the incidence trend analysis, we adjusted Poisson regression models by sex. The dependent variable was the annual incidence, and the explanatory variables were the HPV group (HPV-related or HPV-unrelated subsites), age group (≤39, 40–59, and 60+ years), and time (years). We used as offset the population size according to the age group and year. With the models, we estimated the incidence rates and the annual percent change (APC; 95% confidence interval [CI]) for each segment (i.e., tumor classification and age group). To assess whether the trends were similar in different segments, we assessed contrasts under the general linear hypotheses [32].

Additionally, we investigated the effects of age, calendar year (period), and birth year (cohort) on incidence rates with Poisson regression to measure the relative risk [33,34]. We constructed age-period-cohort models according to 5-year age groups (from 15–19 years to 75+ years) and 5-year calendar periods (1999–2003, 2004–2008, and 2009–2013). The lowest deviance indicated the best-fitted model (p<0.05), and we used R version 3.5.3 (Epi package version 2.3.5) for the analyses.

## Results

Over the entire study period, there were 15,391 new cases of OPC/OCC in São Paulo city. Of these cases, 5,898 occurred in HPV-related subsites (38.3%), and 9,493 occurred in HPV-unrelated subsites (61.7%). There was a marked male dominance in both HPV-related and HPV-unrelated subsites and all anatomical site groups (Table 1). Nevertheless, the proportion of cases in the HPV-related subsites in young individuals aged ≤39 years was 3-fold higher in females than in males (18.0% vs. 5.8%). Likewise, the sex ratio of the incidence decreased more in the HPV-related subsites than in the HPV-unrelated subsites, from 6.2 to 3.5 and 2.4 to 2.1, respectively.

**Table 1:** Descriptive analysis of OPC/OCC by the subsites, sex, and HPV groups.

	TOTAL	SEX		
		Male	Female	Ratio (M:F)
***All OPC/OCC sites***	15,391 (100.0%)	11,538	3,853	3.0
***HPV-related***	5,898 (38.3%)	4,801	1,097	4.4
Base of the tongue	1,287 (8.4%)	1,062	225	4.7
Oropharynx	2,275 (14.8%)	1,915	360	5.3
Tonsils	1,545 (10.0%)	1,189	356	3.3
Waldeyer’s ring, soft palate and uvula	791 (5.1%)	635	156	4.1
***HPV-unrelated***	9,493 (61.7%)	6,737	2,756	2.4
Gums	625 (4.1%)	372	253	1.5
Hard palate	836 (5.4%)	486	350	1.4
Mouth	3,847 (25.0%)	2,810	1,037	2.7
Other parts of the tongue	4,185 (27.2%)	3,069	1,116	2.8

M:F: male:female ratio.

**Fig 1:**
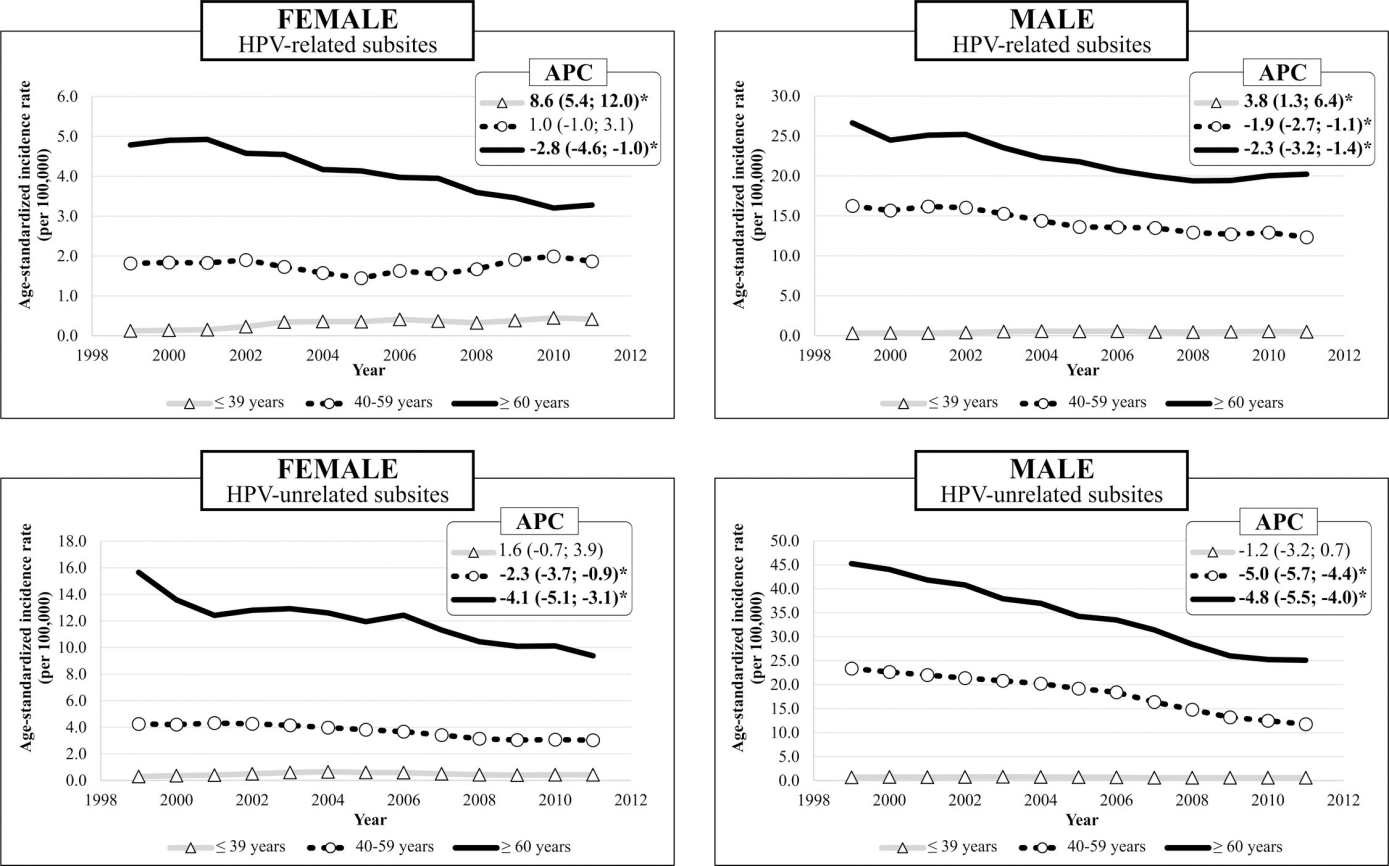
Incidence trends for HPV-related and HPV-unrelated subsites by sex and age group. ^**a**^ APC: annual percent change; *: statistically significant APC. ^a^ For better graph visualization, we applied a simple moving average of 5 years.

In the temporal analysis, incidence trends decreased or remained stable for all anatomical sites, sexes, and age groups (S1 Fig and S2 Fig). For the HPV groups, the incidence rate decreased more sharply in the HPV-unrelated subsites, from 7.4 to 3.3 per 100,000, than in the HPV-related subsites, from 4.1 to 2.6 per 100,000. Moreover, we found a dramatic increase in incidence for HPV-related subsites in young males and females, by 3.8% and 8.6% per year, respectively; in contrast, HPV-unrelated subsites had a decreased incidence rate for most sexes and age groups (Fig 1).

When we compared the inclines (β1) with the contrasts under the general linear hypotheses, we found statistically significant differences among HPV-related and HPV-unrelated subsites for young females (p≈ 0.010) and males (p = 0.033). Additionally, the incidence trends were different between individuals aged ≤39 years and older age groups in both sexes for HPV-related subsites, which indicates a distinct pattern for HPV-related and HPV-unrelated subsites in the young population.

**Fig 2:**
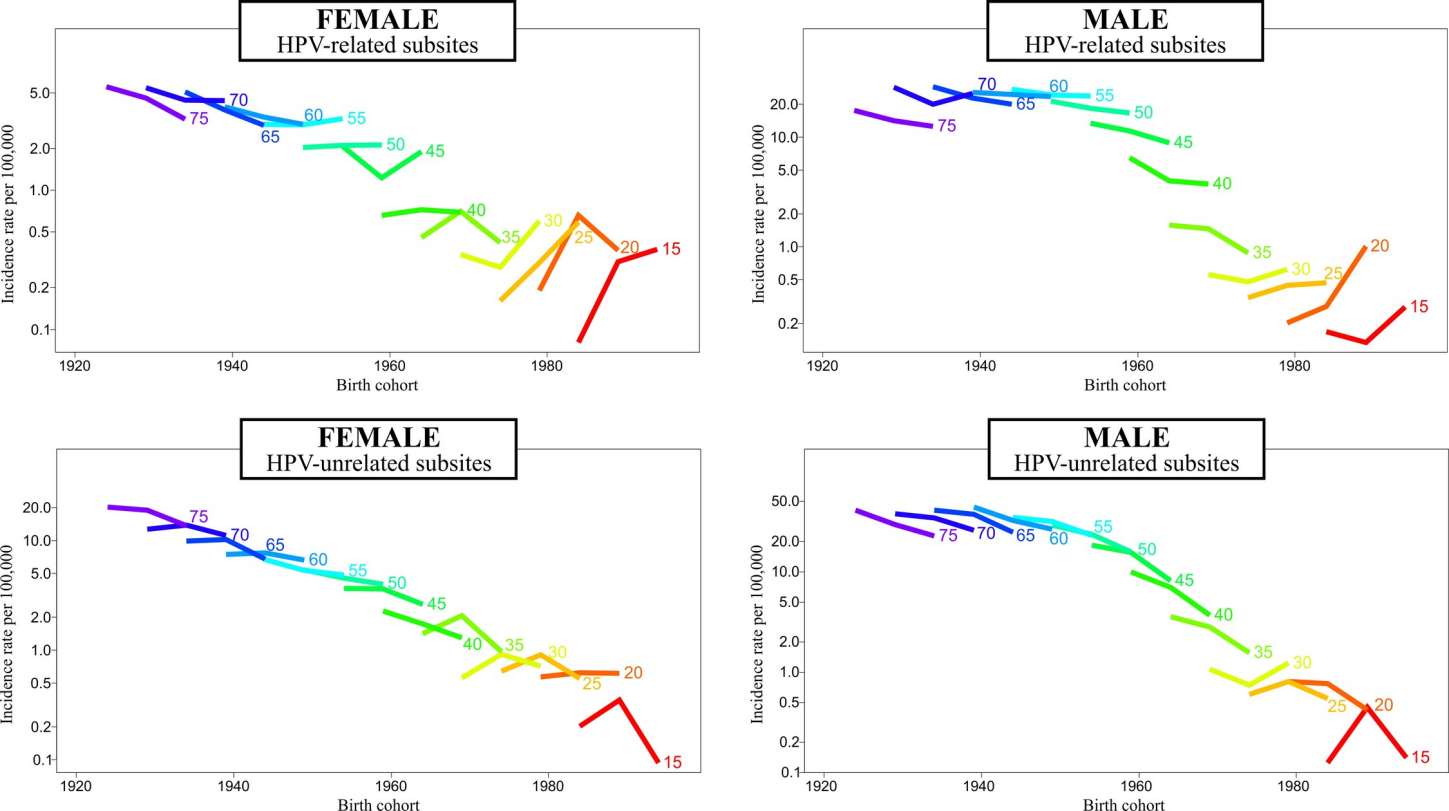
Descriptive birth cohort analysis.

**Fig 3:**
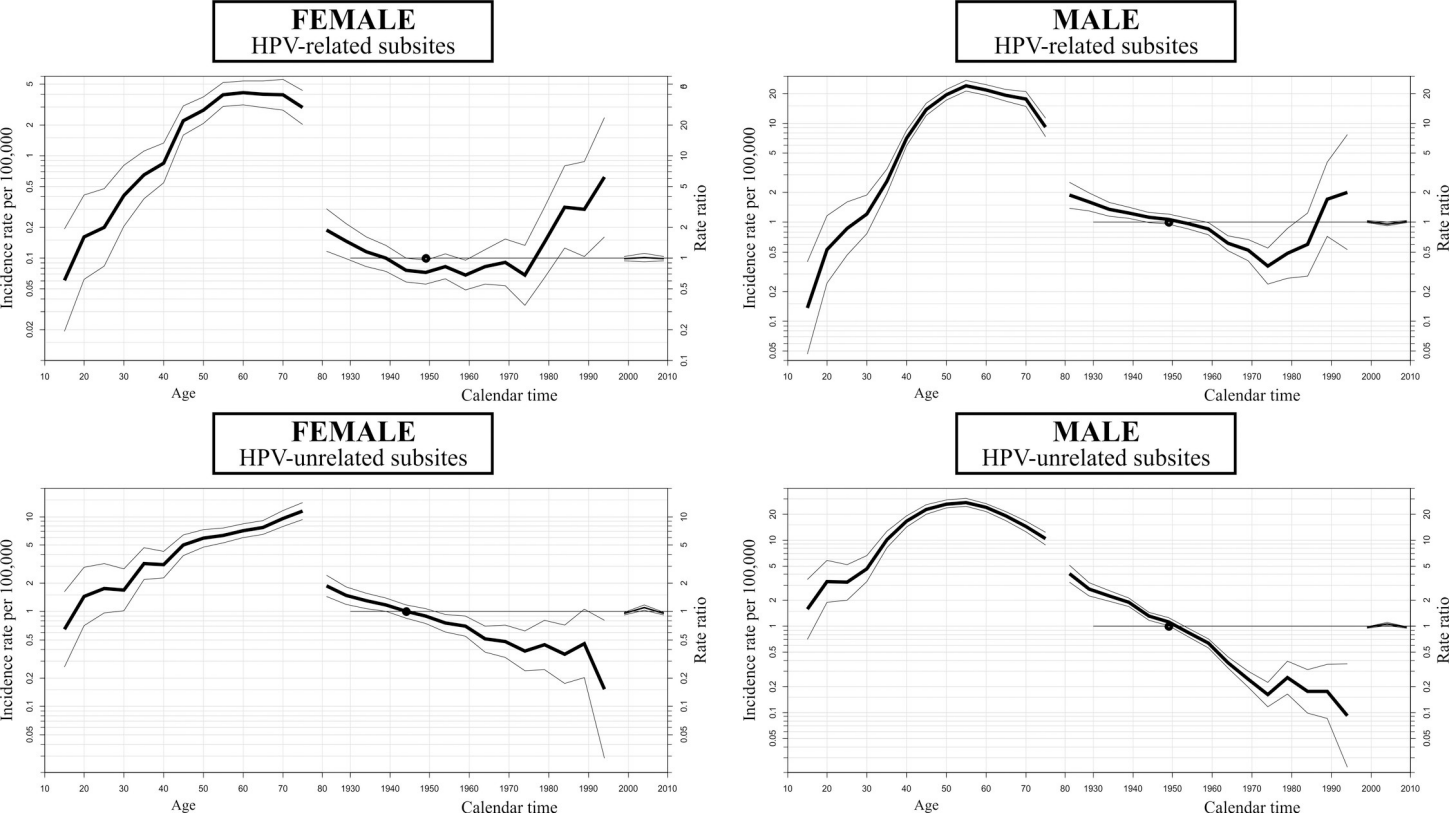
Age-period-cohort effect analysis.

In the age-period-cohort analysis, the incidence rates of HPV-related OPC/OCC increased in the youngest birth cohorts in both sexes, while it decreased in HPV-unrelated subsites (Fig 2). For HPV-related subsites, the risk increased for cohorts born after 1984 in females, with a sharp rise in recent cohorts of males. Conversely, the risk of HPV-unrelated OPC/OCC was decreased in recent birth cohorts in both sexes (Fig 3). In the age-period-cohort models, there was an age-cohort effect in HPV-related subsites and an age-period-cohort effect in HPV-unrelated subsites in both sexes (Table 2).

**Table 2:** Age-period-cohort models presented by sex and the HPV-related and HPV-unrelated subsites.

Model	Female				Male			
	Resid. Df	Resid. Dev	Deviance	p-value	Resid. Df	Resid. Dev	Deviance	p-value
**HPV-related**								
Age	26	43.477			26	88.034		
Age-Drift	25	42.438	1.038	0.308	25	52.329	35.705	<0.001*
Age-Cohort	12	10.832	31.607	*0*.003*	12	15.429	36.900	*<0*.001*
Age-Period-Cohort	11	10.776	0.055	0.814	11	12.023	3.406	0.065
Age-Period	24	42.374	-31.598	0.003*	24	48.994	-36.971	<0.001*
Age-Drift	25	42.438	-0.064	0.800	25	52.329	-3.335	0.068
**HPV-unrelated**								
Age	26	60.580			26	346.440		
Age-Drift	25	30.405	30.175	<0.001*	25	61.740	284.696	<0.001*
Age-Cohort	12	22.483	7.923	0.849	12	18.470	43.270	<0.001*
Age-Period-Cohort	11	13.165	9.318	*0*.002*	11	8.800	9.676	*0*.002*
Age-Period	24	21.008	-7.844	0.854	24	52.130	-43.329	<0.001*
Age-Drift	25	30.405	-9.397	0.002*	25	61.740	-9.618	0.002*

Df: degree of freedom; Dev: deviance

*: statistically significant.

## Discussion

Understanding the HPV pattern over time is critical to support prophylactic interventions focused on diminishing its harmful impacts on populations. For this reason, we performed this study to determine the burden of HPV in OPC/OCC in São Paulo city, which is the largest city in Latin America. Overall, the incidence rates decreased for most OPCs/OCCs in both sexes in all age groups, and particularly in those with HPV-unrelated subsites. However, we identified a dramatically increasing incidence trend for HPV-related OPC/OCC in young females, emphasizing the highest risk in recent birth cohorts.

Our study revealed a striking reduction in the overall incidence trends of OPC/OCC and an age-period-cohort effect in HPV-unrelated subsites. Since the 1990s, the Brazilian tobacco control policy has led to a decrease in smoking [14] and, consequently, tobacco-related cancers. In São Paulo city, the estimated prevalence of smoking decreased in males from 23.6% to 15.6% and in females from 14.6% to 9.8% over thirteen years [35]. Indeed, these data explain our cancer incidence and the lowest risk found in individuals with HPV-unrelated sites in recent generations, which is understandable due to the critical role of tobacco in carcinogenesis [3,36] and the public policy that affects the whole population [14,35].

The lung cancer incidence is decreasing worldwide, while the OPC incidence is increasing, which suggests that HPV infection is a reason for this growth [15]. In this study, the incidence dramatically trended upward in young males and females in HPV-related subsites, particularly in recent cohorts. Our finding is analogous to data from Denmark, which demonstrated an increase in young and middle-aged males and females [37], and France, which demonstrated an increased cumulative risk in females [29]. Conversely, there was a sharp increase in all age groups in Taiwan and the United States (1995–2005) [38]. Additionally, incidence trends increased in older females in England [39], Italy [40], the Netherlands [41], and the United States [42], and there was a negative cohort effect among females in Hong Kong [43]. In summary, these controversial data demonstrate different outbreak periods of HPV infection across populations. Moreover, these results highlight the regional heterogeneity in HPV prevalence, as HPV transmission depends on high-risk sexual behaviors and the virus’s circulation among individuals.

A possible cause for the increased incidence of cancer at HPV-related subsites is the changing sexual behaviors [44]. People who engage in high-risk sexual behaviors are likely to develop cancers in the oropharynx, tonsils, and base of the tongue [45]. Not only did the cases of HPV-related OPC/OCC increase similarly to the rates of genital warts and genital herpes [39], but the HPV prevalence in OPC cases also increased over time. In addition, the incidence of cancers peaked for birth cohorts born between 1943 and 1958 [46], which suggest temporal coherence. Moreover, it is biologically plausible that HPV requires more than ten years to produce a tumor [47]. In Brazil, the median age at first sexual intercourse is 16.5 and 18.5 years in males and females, respectively. Additionally, the occurrence of sexual intercourse before the age of 15 years has increased in females and especially in males, who accounted for 29.6% of men, representing the second-highest global prevalence [48]. HPV is a known cause of cervical cancers [49], and the mortality attributable to this tumor is increasing in Brazilian females aged 20 to 39 years [50]. Additionally, the average age at first diagnosis, 35.4 years, was the lowest among those of all cancer types in females [51]. Under these circumstances, we suppose that changes in the sexual behavior of recent generations have led to an emerging burden of HPV-related diseases in Brazil, as observed in HPV-related OPC/OCC subsites in young individuals in our investigation.

Another potential reason for our findings is the prevalence of alcohol and tobacco use by the individuals with cancer in HPV-related subsites. Previous studies have reported an HPV infection rate of 6.2% in a healthy Brazilian population [52], and the detection rate of HPV16 was 4.1% in OPC patients from São Paulo [18]. Conversely, in São Paulo city, 79.6% of the subjects aged between 16 and 25 years reported high-risk sexual behavior. Consequently, HPV was detected in 52% of samples from the uterine cervix, penis, and scrotum, of which 38.8% were positive for viral strains with high-risk of progression to cancer [53]. Furthermore, smoking prevalence decreased in females [35]. For these reasons, it is plausible that alcohol and tobacco use do not fully clarify the increasing risk observed in young individuals.

Although our results are not sufficiently robust to indicate a viral epidemic in São Paulo city, they highlight the need to monitor the burden of these cancers in young populations. Considering that cancer is a time-dependent disease, we expected stable trends for individuals aged ≤ 39 years, but we found an upward trend in HPV-related OPC/OCC in young individuals. In addition, the remarkable decrease in smoking may be masking the increasing incidence in the HPV-related subsites, as this tumor classification occasionally includes tobacco-related cancers. For this reason, our data suggest that a dramatic increase in HPV-related OPC/OCC in its initial phase is occurring in the city of São Paulo. Indeed, this is a public health concern due to high survival rates and its potential magnitude, which impairs patients’ quality of life and the healthcare and social security systems, particularly in developing countries, such as Brazil.

Our study had limitations that are important to its interpretation. As in other countries [25,46], cancer registries do not gather data on positive HPV DNA within tumors. Accordingly, we used anatomic sites as a proxy to categorize OPCs/OCCs as those occurring in either HPV-related or HPV-unrelated subsites. Moreover, we had no information on alcohol and tobacco use and high-risk sexual behaviors in the subjects. However, several investigations had similar limitations, especially regarding tumor classification [8,11,12,24,25,37,46,54–56]. Furthermore, this grouping had analogous results to laboratory data results [57], suggesting it is a reliable method since HPV testing is not a standard procedure in cancer registries.

This population-based study had strengths including its generalizability and limited selection bias, which allowed us to demonstrate the emerging risk of HPV-related OPC/OCC in young males and females, whereas the risk in HPV-unrelated subsites sharply decreased. For these reasons, our data support the HPV vaccination program and the continuity of the Brazilian tobacco control policy, which has had positive effects on OPC/OCC incidence in São Paulo city. Furthermore, it is crucial to continue to specifically monitor the incidence of HPV-related OPC/OCC and to investigate vaccine effectiveness in the oropharyngeal and oral cavity subsites in the long term. Moreover, cancer control programs should broaden screening coverage in young subjects to prevent sexually transmitted infections and HPV-related cancers to minimize the increasing risk.

## Supporting information

S1 Fig OCC/OPC incidence trends by anatomic site and sex (1997–2013). [A]: Base of the tongue; [B]: oropharynx; [C]: tonsils; [D]: Waldeyer’s ring, soft palate and uvula^1^; [E]: gums; [F]: hard palate; [G]: mouth; and [H]: other parts of the tongue.^a,b^ APC: annual percent change; *: statistically significant APC (95% CI). ^a^ For better graph visualization, we applied the simple moving average of 5 years. ^b^ We analyzed these data with joinpoint regression models. ^1^ As there were 11 cases of cancer in Waldeyer's ring, they were combined with the cases of cancer in the soft palate and uvula.
(TIF)

S2 Fig OCC/OPC incidence trends by anatomic site (1997–2013). [A]: Base of the tongue by age groups; [B]: oropharynx by age groups; [C]: tonsils by age groups; [D]: Waldeyer’s ring, soft palate and uvula^1^ by age groups; [E]: gums by age groups; [F]: hard palate by age groups; [G]: mouth by age groups; and [H]: other parts of the tongue by age groups.^a,b^ APC: annual percent change; *: statistically significant APC (95% CI). ^a^ For better graph visualization, we applied the simple moving average of 5 years. ^b^ We analyzed these data with joinpoint regression models. ^1^ As there were 11 cases of cancer in Waldeyer's ring, these cases were combined with the cases of cancer in the soft palate and uvula. **#** There were insufficient cases for analysis.
(TIF)

S3 Fig [A]: Incidence trends for OCC/OPC according to HPV groups; [B]: incidence trends for OCC/OPC by sex and HPV groups; [C]: incidence trends for HPV-related OCC/OPC by age groups; and [D]: incidence trends for HPV-unrelated OCC/OPC by age groups.^a,b^ APC: annual percent change; *: statistically significant APC (95% CI). ^a^ For better graph visualization, we applied a simple moving average of 5 years. ^b^ We analyzed these data with joinpoint regression models.
(TIF)
