# Supplementary figures and images for: The emerging risk of oropharyngeal and oral cavity cancer in HPV-related subsites in young people in Brazil
Source: PLoS One. 2020 May 14;15(5):e0232871. doi: 10.1371/journal.pone.0232871 (PMC7224475; doi:10.1371/journal.pone.0232871)

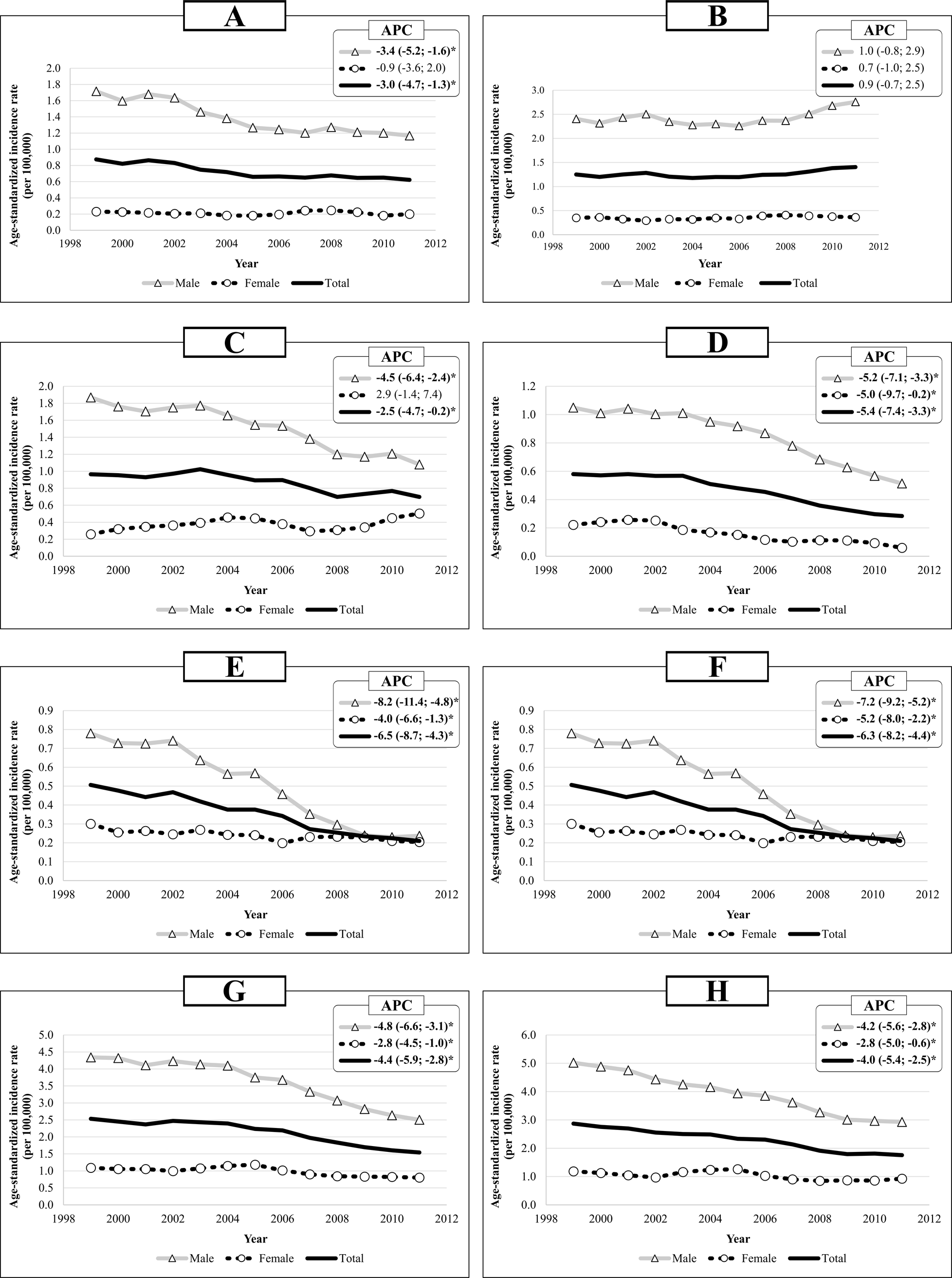

Supplement: S1 Fig — [A]: Base of the tongue; [B]: oropharynx; [C]: tonsils; [D]: Waldeyer’s ring, soft palate and uvula1; [E]: gums; [F]: hard palate; [G]: mouth; and [H]: other parts of the tongue.a,b APC: annual percent change; *: statistically significant APC (95% CI). a For better graph visualization, we applied the simple moving average of 5 years. b We analyzed these data with joinpoint regression models. 1 As there were 11 cases of cancer in Waldeyer's ring, they were combined with the cases of cancer in the soft palate and uvula. (TIF) [file pone.0232871.s001.tif]

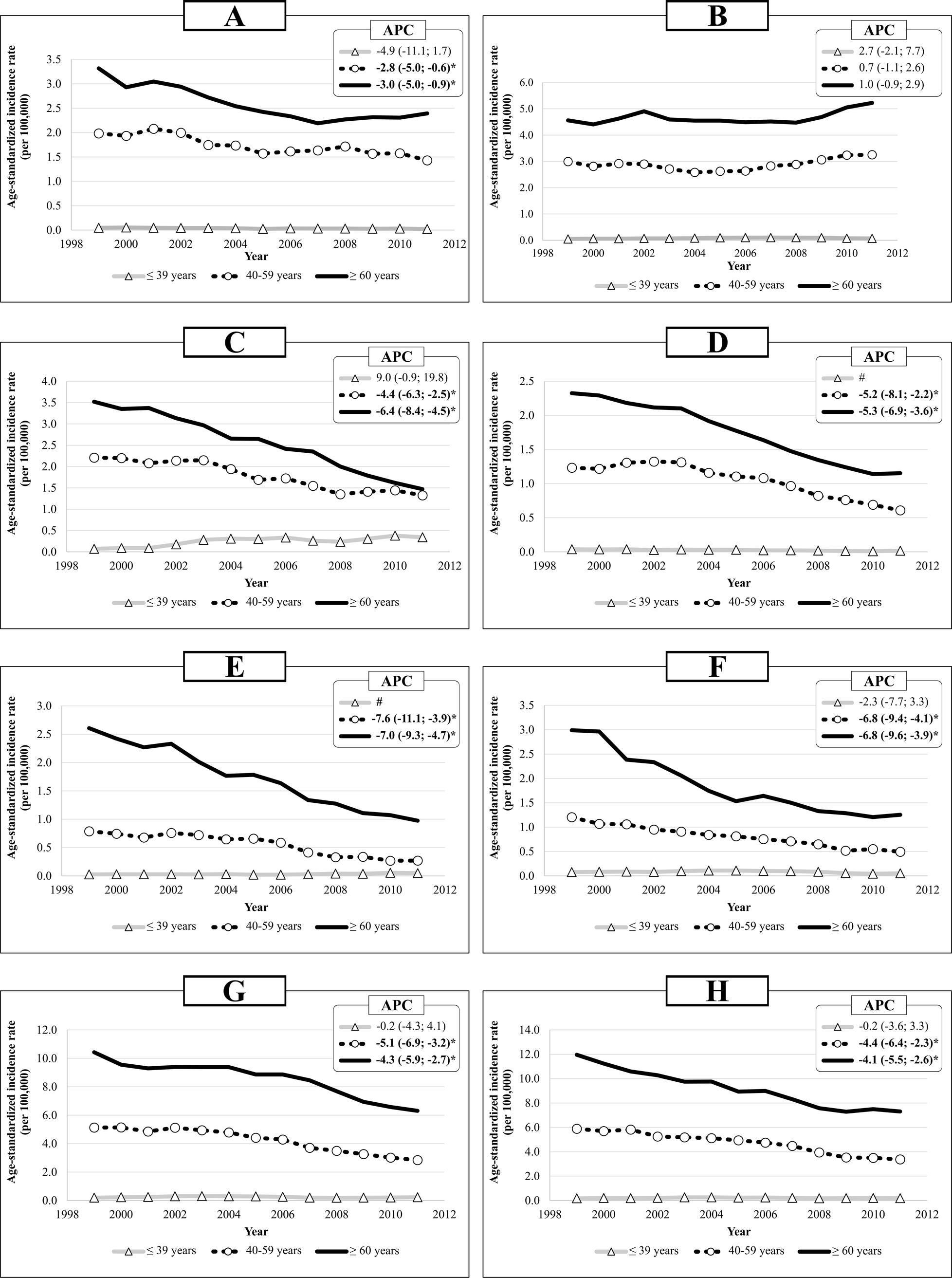

Supplement: S2 Fig — [A]: Base of the tongue by age groups; [B]: oropharynx by age groups; [C]: tonsils by age groups; [D]: Waldeyer’s ring, soft palate and uvula1 by age groups; [E]: gums by age groups; [F]: hard palate by age groups; [G]: mouth by age groups; and [H]: other parts of the tongue by age groups.a,b APC: annual percent change; *: statistically significant APC (95% CI). a For better graph visualization, we applied the simple moving average of 5 years. b We analyzed these data with joinpoint regression models. 1 As there were 11 cases of cancer in Waldeyer's ring, these cases were combined with the cases of cancer in the soft palate and uvula. # There were insufficient cases for analysis. (TIF) [file pone.0232871.s002.tif]

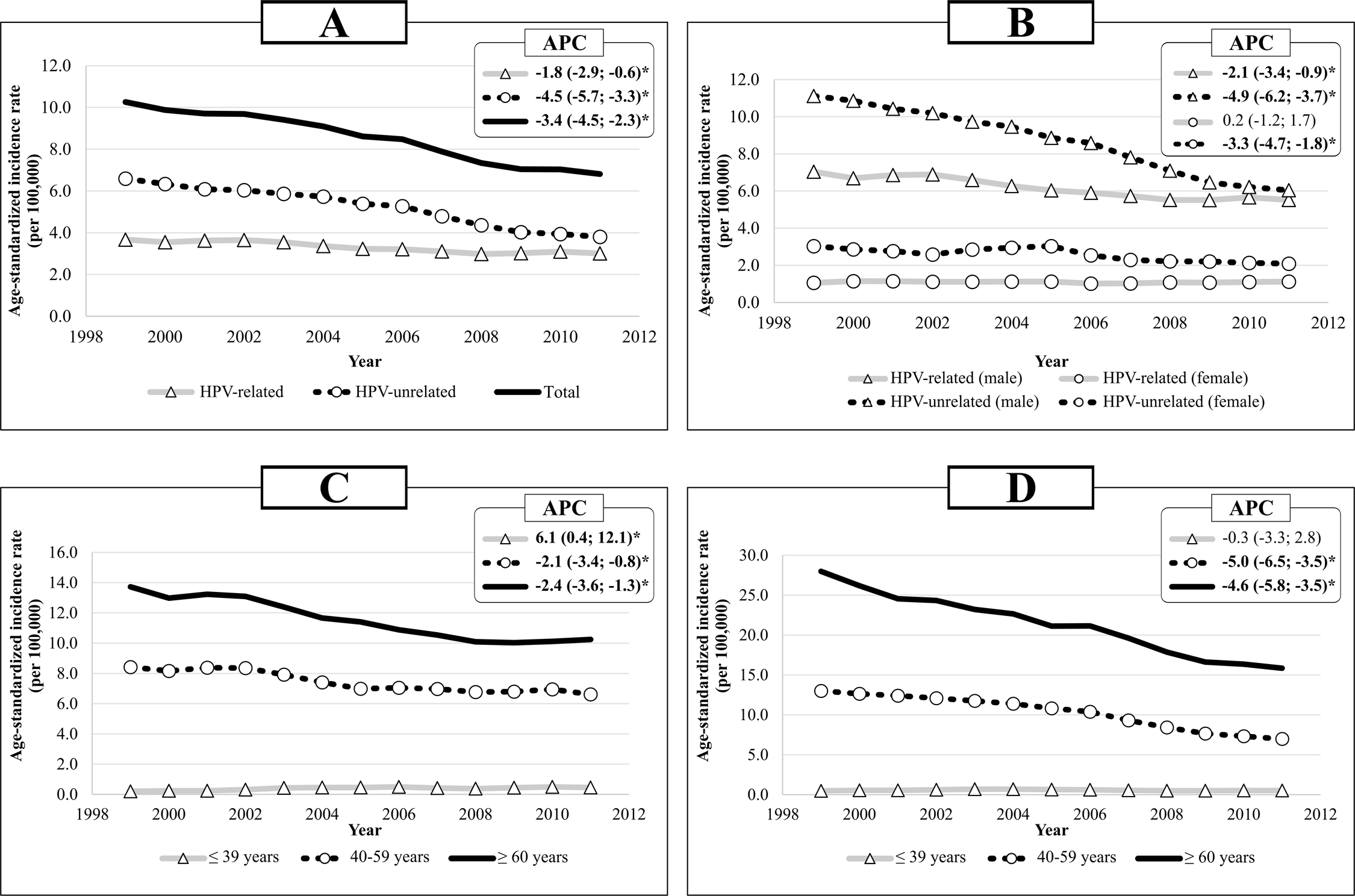

Supplement: S3 Fig — [A]: Incidence trends for OCC/OPC according to HPV groups; [B]: incidence trends for OCC/OPC by sex and HPV groups; [C]: incidence trends for HPV-related OCC/OPC by age groups; and [D]: incidence trends for HPV-unrelated OCC/OPC by age groups.a,b APC: annual percent change; *: statistically significant APC (95% CI). a For better graph visualization, we applied a simple moving average of 5 years. b We analyzed these data with joinpoint regression models. (TIF) [file pone.0232871.s003.tif]
